# Hip Joint Disease—How to Treat and How to Know It*Read before the Jackson County Medical Society, October, 1898.

**Published:** 1898-12

**Authors:** Herman E. Pearse

**Affiliations:** Kansas City, Mo.; Formerly Professor of Anatomy of Kansas City Medical College and to the Woman’s Medical College, Consultant to the Kansas City, Fort Scott & Memphis Hospital, and the Woman’s Hospital, Editor of Medical Index, etc.


### HIP JOINT DISEASE—HOW TO TREAT AND HOW TO KNOW IT.*

By HERMAN E. PEARSE, M.D., Kansas City, Mo.

Formerly Professor of Anatomy of Kansas City Medical College and to the Woman's Medical College, Consultant to the Kansas City, Fort Scott & Memphis Hospital, and the Woman's Hospital, Editor of Medical Index, etc.

About our streets are frequently seen the withered, distorted limbs of those who have fallen victims to the ravages of chronic joint disease; the most distressing and most noticeable being those unfortunates who have one withered lower limb, and one sound one, a crutch and a way of swinging themselves along upon it, handicapped forever in the race for existence, debarred from most pursuits requiring activity or grace and too often crowded to the wall unless shielded and helped by kind relatives or sheltered by some institution which provides a place for cripples. From this complete picture to one of health, we find many gradations, all offering more or less impediment in the way of the unfortunate individual ever securing the full share of enjoyment due to an American in the full possession of his powers and faculties. These unfortunates in almost every instance are the victims of the ravages of "hip joint disease"—morbus coxarius—a chronic tuberculous inflammation at, or immediately about the epiphysis of the upper end of the femur. Every one of them, barring an insignificant number of weaklings, might have had two feet to stand upon instead of one and a crutch, could only the chance have been given nature to right the wrong suffered by the early invasion of the tissues by inflammation.

I undertake this paper in the hope that something I may say will make it easier for our medical men to show to the parents of those in their care how necessary, nay how imperative, is the call for early and intelligent and persistent action when this trouble threatens; for I believe the responsibility for a cripple from hip joint disease rests most often upon the head of him and of her who loves the poor child best; upon the heads of the father and mother, who can not see or will not see how urgent is the need for action. The doctor is not so often at fault. And yet there are cases sent to us here at our Kansas City hospitals that have never been recognized by the doctor in charge, although it would seem that this disease whose correct treatment has been known and practiced for thirty years should be so familiar to every tyro in the medical profession that a consultant would only have to confirm the diagnosis correctly made and the treatment correctly instituted in every case. Unfortunately this is not always so.

In teaching my students in anatomy the pathological an-

*Read before the Jackson County Medical Society, October, 1898.

atomy of the groin, I have been accustomed to tell them that in the case of a "lump in the groin" without definite history to the contrary, it was a case of "hernia against the field" in the diagnois—consider it hernia until they could prove it otherwise. So I would say to those higher and more earnest students, my colleagues in the medical profession whom I have the honor to address to-day, that, in case of a limping child whose foot is well or in case of one who has persistent pain in the upper leg or knee, it is a case, in diagnosis, of destructive bone disease against the field. In the majority of cases this is a hip joint disease; otherwise it is apt to be suppurative osteo-myelitis.

The most ingenious excuses are invented to account for such commencing disability. "The child has a strain," "the child favors the right leg because it's kind of drawn," "he bumped that hip six months ago; it was sore awhile and he never learned to use it right again," as if a trivial trouble of the muscles could so interfere with the play impulses of childhood as to restrict motion! No. A limping child, one with a leg which it "favors," one who cries out with ill-defined pain in the leg or hip, or knee or thigh, is a probable victim of destructive bone disease. Let us look for it upon suspicion, let us find it, if present, or know of its absence, and let us treat it upon rational principles up to successful cure while yet we can, convincing by our demonstrations and our positive knowledge the wavering parent who "only wants some liniment to take out the soreness." I remember one of my patrons who sent a boy to me with a note to "please examine Johnnie's leg and send her fifteen cents' worth of something to cure it with."

In referring to the stages of hip joint disease in this paper, I mean by first stage the condition of entrance of inflammation (almost invariably tuberculosis) into the tissues about the head of the bone—at the epiphysis in most cases—and the consequent irritation and new formation. By second stage, the development of arthritis with its effusion and tissue growths; and by the third stage, the breaking down of tissue and the greater or less disorganization and destruction of the joint and the beginning of efforts at repair, which often fail and often produce severe deformity. Thirty years ago the mortality from this disease was above thirty-five per cent., and a goodly majority of those that lived were hopeless cripples and weaklings. Now, thanks to rest, extension and antiseptic surgery, it is below five per cent. Marsh says of hip disease: "Whatever the origin, once it commences to advance, it soon involves not only the soft structures of the joint, but frequently also both the upper end of the femur and the floor of the acetbulum. Owing to the cancellous structure of the bones, the inflammatory process commonly ends in caries, rather than necrosis; and though sequestra are sometimes found, they are seldom much larger than a nut, and consist of soft fragments easily broken down in the discharge. Cases, however, are not rare in which, as the result of acute inflammation at the junction of the epiphysis with the neck, the whole head of the femur, or what remains of it, becomes detached from the neck, and is found lying in the interior of an abscess, or in the cavity of the joint. In extensive disease of the acetbulum, sequestra may be found, but they are usually small and friable. Ultimately the head and neck of the femur, as well as the rim of the acetbulum having been absorbed, the upper end of the femur is displaced upwards and backwards on the dorsum ilii, and accompanying this there is usually an increase of deformity in the direction of flexion and adduction of the limb. In the worst cases the bones become very extensively involved. Chronic inflammation (osteomyelitis) spreads down to a considerable distance along the medullary tissue of the femur, and leads to widespread necrosis. In other instances, the floor of the acetabulum becomes so extensively carious, that the cotyloid cavity is entirely destroyed."

Says the same author: "Lameness arises either because pain restrains the patient from the free use of the limb, or because the limb is fixed in some abnormal position which, however, varies with the different stages of the disease. These two causes of lameness often exist together. As, however, each posture produces its peculiar limp, as the limp will vary with the amount of pain, and as various kinds of lameness are often due to affections of the spine or other parts, it will be understood that there is *no form that is in the least characteristic of hip disease. The symptom is valuable as indicating that something is amiss; but, taken alone, it is of no diagnostic value.* (Italics mine.)

"Pain varies greatly in its amount. Sometimes, from first to last, it is so slight as to mask the disease. At others it is severe and persistent. The following nerves send twigs to the hip joint: The anterior crural, branches from which usually, but not constantly, pierce the front of the capsule; some from the sciatic and sacral plexus enter behind; and a twig from the obturator reaches the interior through the cotyloid notch. From these various nerves other peripheral offsets are supplied to various parts of the limb below. Branches from the anterior crural enter the knee through the front, and from the obturator through the back of the capsule; and other twigs from the obturator end in the inner side of the thigh (in the saphenous plexus); occasionally a branch runs down along the inner side of the leg. This nerve distribution is alluded to in order to explain how it is that pain may be felt either in the hip joint itself, or in the knee, the inner side of the thigh or inner aspect of the leg. Its occurrence in the parts of the limb below the hip is an example of the reference of pain to the longest peripheries of sensory nerves.

"It is well known that when the hip is affected pain may be so entirely limited to the knee as to lead to an oversight as to the real situation of the disease. Pain referred to the inner side of the thigh or the leg is not very commonly met with, yet distinct examples of it are now and then to be seen. It must be remembered that pain is occasionally referred to the knee and other parts of the limb in several affections besides the hip disease; e. g., in cariesof the lumbar spine, inflammation of the sacro-iliac joint, and abscess in the pelvis, or in Scarpa's triangle; so that this symptom is in itself in no way conclusive as to the presence of disease of the hip. It becomes valuable only when it is found conbined with other signs. Sometimes pain is so slight that parents will not believe that the joint is affected; but its absence must not throw the surgeon off his guard. The severe pain which recurs whenever the patient drops off to sleep, and which leads to the well-known night screams, is produced by the sudden pressure together of the articular surfaces during violent contraction of the muscles around the joint, resulting from reflex irritation." This from one of the highest authorities on bone disease in the profession.

Of these various stages, one, the first, requires exceptional care in diagnosis. Leaving to text-books the various postures and their inferences and the minor points of value in determining the exact stage and condition, I will say that in this doubtful first stage—the most difficult to assert, the hardest to diagnosis with assurance—the following tests will show its presence; for as Valette has said: "There can be no hip disease if motions at the hip joint are perfect and unrestricted."

First, flexion: bring the heel to the bullocks; carry the knee towards the belly.

Second, abduction: while the leg is flexed upon the thigh, carry the knee outward until it rests upon the bed.

Third, abduction: while the leg is flexed upon the thigh, carry the knee across the well leg.

Fourth, extension: while the back rests on a firm mattress, extend the leg until the popliteal space rests against the mattress in the same plane.

Fifth, rotation.

If these motions can be made without finding muscular rigidity, there is no hip disease. If they can not, we must suspect hip disease until we can account for the rigidity. Given the diagnosis, there are three cardinal lines of treatment to be followed:

First, rest for the limb, by recumbent posture and extension until all muscular spasm ceases during rest.

Second, build up the patient by air, bathing, hygiene, food, milk and medicine to the highest point obtainable.

Third, apply good, sound surgical principles to the collections of pus that may be present or may form—leaning always strongly toward conservation in operating; open abscesses only when it is clearly apparent they will not be reabsorbed—performing operations upon bone with the greatest reluctance, or amputating when forced to by the exigencies of the case and being guided by the same rules that should ever guide a careful surgeon. Here is no place for bold operating, no place for extensive cutting. Operate only when good surgical sense demands it, not otherwise. Wonders are accomplished in this particular disease, by good rest and delay.

The extension I employ is the "Buck's extension," put on as follows: Cut a strip of adhesive plaster three inches wide and long enough to reach well above the knee on both sides of the leg, leaving a free loop of four to six inches below the sole of the foot. To prevent pressure upon the ankle joint, a piece of thin board five inches long and two inches wide, with a hole in the center, is fastened in this loop and the edges of the plaster brought over the edges of the wood. A little cotton should be put over each malleolus. A roller bandage is applied over the entire limb after the plaster has been smoothly laid along the leg. From three to seven pounds of sand are put in bag; a long, hard cord tied to the neck of the bag, carried over a pulley at the foot of the bed and tied into the little foot-board in the extension straps. The pulley is best set in a piece of wood that can be lowered a little each day. From four to six days are needed to relax a leg, and it then commences to straighten; gradually this pulley is lowered, about one-half inch each day, until the femur is straight with the axis of the body and the two limbs equal in length. Counter extension is obtained by two or three bricks under the foot-posts of the bed. The surgeon's judgment must determine when to open a joint—when to apply a brace after the extension has quieted the acute symptoms, and when to apply constitutional treatment and what that must be.

As to abscess formation: "We have three grades of abscess, so to speak, to bear in mind: First, those which occur in the full tide of the acute inflammatory process.

"Second, those which form when the inflammatory process is subacnte, or in dying away.

"Third, those which are developed when all disease has subsided, and when necrotic products of bygone disease have to be cast out. The results of treatment correspond very closely with these different conditions. In the first kind, the mere evacuation of the matter already formed, although it may notably check, can not be expected suddenly to arrest the process of suppuration. Nor does it do so; but what is gained by interference is this: that when the abscess is opened, as the drainage tube provides a passage for the escape of pus as it is formed, the cavity soon contracts to the dimensions of a mere sinus, through which, day by day, each day's secretion quietly flows away; so that, in other words, a sinus for the free escape of matter as it forms is substituted for a rapidly increasing abscess in the limb. The period during which this sinus continues to discharge, and the amount of pus which flows from it, will, of course, depend upon the amount and persistence of the disease by which it is produced. In the second form, the abscess, evacuated at the operation, remains empty, the sack contracts, and in the course of a fortnight or three weeks the wound appears to be finally healed. Subsequently, however, sometimes only after the lapse of several weeks or even months, it is found that some re-accumulation is taking place, and at last the wound reopens or must be opened, and a small quantity of pus escapes, after which final closure takes place; or if this does not occur, a small sinus remains for a few weeks discharging occasionally, and then, when the last remnants of the original disease have died out, it soundly heals. In the third variety, the emptying of the accumulation is followed by immediate closure, no more matter being formed; and within a fortnight or a month the abscess has completely disappeared, the tissues of the limb have regained their natural condition, and all that remains is the clean and firm scar of the incision.

"For my own part, I am very strongly convinced that the best course is to open any abscess that forms about the hip as soon as it is detected. But it is best in all instances to ensure that the limb has been at perfect rest for at least a week or ten days before operating. If a child has an abscess when first seen, and before the rest-treatment has been entered upon, interference with the abscess should be postponed for a short time till the circulation has become quiet, and the vessels surrounding the area of inflammation have become less engorged.

"There is no doubt that under continuous rest abscesses, sometimes of large size, holding at least six ounces, may be absorbed. It is, however, probably better that matter should be let out by incision. Sir James Paget has recorded instances in which abscess cavities that had been emptied by absorption of their contents have, either from some disturbance of the general health, over-exertion, or other cause, suddenly refilled, and I have seen several similar instances. Healing is more sound and complete when pus has been evacuated."

But again I must say that this brilliant surgery must not be invoked until spasm is reduced and until recovery without it is clearly not to be hoped for.—*Kansas City Medical Index.*

